# Green Synthesis and Characterization of Silver Nanoparticles from *Tinospora cordifolia* Leaf Extract: Evaluation of Their Antioxidant, Anti-Inflammatory, Antibacterial, and Antibiofilm Efficacies

**DOI:** 10.3390/nano15050381

**Published:** 2025-03-01

**Authors:** Vijaya Durga V. V. Lekkala, Arun Vasista Muktinutalapati, Veeranjaneya Reddy Lebaka, Dakshayani Lomada, Mallikarjuna Korivi, Wei Li, Madhava C. Reddy

**Affiliations:** 1 Department of Genetics and Genomics, Yogi Vemana University, Kadapa 516005, India; vijaya@yvu.edu.in (V.D.V.V.L.); dlomada@yvu.edu.in (D.L.); 2 Department of Biotechnology and Bioinformatics, Yogi Vemana University, Kadapa 516005, India; m.arunvasista@gmail.com; 3 Department of Microbiology, Yogi Vemana University, Kadapa 516005, India; lvereddy@yvu.edu.in; 4 College of Physical Education and Health Sciences, Zhejiang Normal University, Jinhua 321004, China

**Keywords:** *Tinospora cordifolia*, nanotechnology, green synthesis, free radical scavengers, antimicrobial activity

## Abstract

The use of metal nanoparticles is gaining popularity owing to their low cost and high efficacy. We focused on green synthesis of silver nanoparticles (AgNPs) using *Tinospora cordifolia* (Tc) leaf extracts. The structural characteristics of Tc nanoparticles (TcAgNPs) were determined using several advanced techniques. Pharmacological activities, including antioxidant, anti-inflammatory, and antibacterial properties, were evaluated through in vitro studies. In the results, the change in sample color from yellow to brown after adding silver nitrate revealed the synthesis of TcAgNPs, and the UV–visible spectrum confirmed their formation. X-ray diffraction studies showed the presence of reducing agents and the crystalline nature of the nanoparticles. Fourier-transform infrared spectra revealed the existence of essential secondary metabolites, which act as reducing/capping agents and stabilize the nanoparticles. The size of the TcAgNPs was small (range 36–168 nm) based on the measurement method. Their negative zeta potential (−32.3 mV) ensured their stability in water suspensions. The TcAgNPs were predominantly spherical, as evidenced from scanning electron microscopy and transmission electron microscopy. Atomic absorption spectroscopy data further revealed the conversion of silver nitrate into silver nanoparticles, and thermogravimetric analysis data showed their thermal stability. The TcAgNPs showed significant DPPH/ABTS radical scavenging ability in a concentration-dependent manner (25–100 µg/mL). Membrane lysis assays showed an effective anti-inflammatory activity of the TcAgNPs. Furthermore, the TcAgNPs showed potent antibacterial effects against multidrug-resistant bacteria (*Pseudomonas aeruginosa*, *Klebsiella pneumonia*, *Escherichia coli*, and *Staphylococcus aureus*). The TcAgNPs treatment also exhibited antibiofilm activity against bacterial strains, in a concentration-dependent manner. Our findings demonstrate the structural characteristics of green-synthesized TcAgNPs using advanced techniques. TcAgNPs can be developed as potential antioxidant, anti-inflammatory, and antibacterial drugs.

## 1. Introduction

Nanotechnology offers a wide range of opportunities for researchers to interact with complex biological functions at the cellular and molecular levels. Due to their unique physical and chemical properties, nanoparticles (NPs) are used as potential drug delivery agents to treat cancer, autoimmune diseases, cardiovascular disease, and diabetes [1,2]. Nanoparticles offer many opportunities to use them for various medical purposes [3]. Green or biosynthesis of NPs is eco-friendly [4], in which plant extracts act as reducing agents [5]. The technique of green synthesis is non-toxic, cost-effective, and more stable than other biological methods. Green-synthesized NPs show various biological and catalytic activities [6]. Noble metals exhibit unique properties because of their morphology, size, and distribution, and they are broadly used in the preparation of NPs [7]. Silver nanoparticles (AgNPs) have been extensively studied in many research areas, owing to their exclusive biological properties [8]. Dating back to 1000 BC, silver has been used as a medicating agent to treat burns and wounds, signifying its medicinal properties. In the 1960s, silver solution (0.5% silver nitrate) was used to treat burns (without interfering with epidermal proliferation) and was reported to have antibacterial properties against *P. aeruginosa*, *S. aureus*, and *E. coli* [9]. Earlier studies demonstrated the antioxidant, anti-inflammatory, antibacterial, antibiofilm, anticancer, and wound-healing properties of AgNPs [10,11,12,13]. Since ancient times, in Ayurvedic medicine, silver particles known as “Rajatha Bhasma” (calcined silver ash) have been widely used in medicinal formulations to treat various ailments [14]. Synthesis of AgNPs through chemical methods is harmful because the use of hazardous chemicals, intense heat, and pressure is redundant and cumbersome. Instead, green synthesis of AgNPs has excellent stability compared to chemical methods [15]. AgNPs synthesized from plant extracts are safe and utilize a wide range of metabolites for promoting the bio-reduction of silver to silver ions [16].

*Tinospora cordifolia* (Tc) belongs to the “Menispermaceae” family and is native to the tropical areas of India, Sri Lanka, and Myanmar. *T. cordifolia* is a large, genetically diverse, and deciduous climbing herb with greenish-yellow flowers [17,18]. Vernacularly, *T. cordifolia* is known as *Giloy*, *Amrita*, *Guduchi* (Sanskrit), *Tippa Teega* (Telugu), and heart-leaved moonseed. Traditionally, *T. cordifolia* extracts have been widely used to treat several ailments [18]. This medicinal plant is considered to be one of the essential herbs of the Indian Systems of Medicine (ISM) [19]. As a climber, *T. cordifolia* grows over hedges and small trees, and it is known for its antispasmodic, antipyretic, antineoplastic, hypolipidemic, hypoglycemic, immune-modulating, and hepatoprotective properties [18,20]. Several biologically active compounds, including alkaloids, diterpenoids, glycosides, steroids, sesquiterpenoids, phenolics, polysaccharides, and aliphatic compounds, have been found in various parts of the plant [21,22]. The medicinal properties of these compounds enable its potential applications in clinical research [19,21]. However, the pharmacological activities of *T. cordifolia* leaf extracts in the presence of metal ions are not yet fully documented. The hybrid material is formed by the metallic nanoparticles, stabilized by the plant compounds, and deposited on the metallic surface of the particles.

In a previous study, Selvam et al. biosynthesized AgNPs using *T. cordifolia* leaf extracts, and they showed antibacterial activity against *Staphylococcus*, *Klebsiella*, and *Bacillus* species. However, they did not address the concentration-dependent antibacterial effect of AgNPs [12]. Ghosh et al. reported the antibiofilm activity and minimum inhibitory concentration (MIC) of TcAgNPs against only *Staphylococcus aureus*, and not with other bacterial species [5]. A recent study emphasized the zone of inhibition of TcAgNPs against *Salmonella typhi*, *P. aeruginosa*, *Enterobacter aerogenes*, and *S. aureus*; however the MIC values, antibiofilm effects, and antioxidant activities with different concentrations of TcAgNPs were not reported [23]. In addition, these studies did not report the detailed characterization of TcAgNPs with advanced techniques like thermogravimetric analysis (TGA) and atomic absorption spectroscopy (AAS). On the other hand, the reported antibacterial activity is not against multidrug-resistant (MDR) bacteria, and the effective concentration of TcAgNPs is yet to be documented. MDR bacteria are deadly pathogens whose spread poses a serious threat to human health [24]. Therefore, the novelty of this study is to determine the structural characteristics of TcAgNPs, and to explore the antibacterial and antibiofilm activities with different concentrations of TcAgNPs against MDR bacteria. Another novel element is to reveal the in vitro antioxidant and anti-inflammatory properties of various concentrations of TcAgNPs.

In the present study, we synthesized AgNPs using *T. cordifolia* leaf extracts (TcAgNPs). The physical and chemical properties of TcAgNPs, including size, shape, morphology, stability, and structure, were determined through X-ray diffraction (XRD), UV–visible spectroscopy, Fourier-transform infrared (FTIR), dynamic light scattering (DLS), zeta potential, scanning electron microscopy (SEM) coupled with energy-dispersive X-ray (EDX), transmission electron microscopy (TEM), TGA, and AAS analyses. The antioxidant activity of the TcAgNPs was determined by DPPH and ABTS radical scavenging assays. The anti-inflammatory activity of the TcAgNPs was evaluated by membrane lysis assays. The antibacterial activity of the TcAgNPs was evaluated by the disc diffusion method, MIC, and minimum bactericidal concentration (MBC) assays against MDR bacteria, and the antibiofilm activity was evaluated by the microtiter plate method.

## 2. Materials and Methods

### 2.1. Collection of T. cordifolia Leaves and Preparation of Extracts

Fresh leaves of *T. cordifolia* were collected locally from the Botanical Garden of Yogi Vemana University, Kadapa, Andhra Pradesh, India. The leaves were repeatedly washed with double-distilled water (dH_2_O) and allowed to dry in the shade (without exposure to sunlight) at room temperature. The dried leaves were taken and coarsely ground in a sterilized mixer grinder. The coarse powder was stored in a Ziplock bag at 4 °C. Then, 5 g of powder was boiled in 100 mL of sterile distilled water on a hot plate magnetic stirrer for 60 min, and then Whatman Grade 1 filter paper was used to filter the contents. The resulting extract was collected and stored at 4 °C for the experiments.

### 2.2. Green Synthesis of AgNPs from T. cordifolia

Green synthesis of AgNPs was performed by mixing *T. cordifolia* leaf extract with various concentrations of silver nitrate (AgNO_3_) (GR Merck, India) solution, ranging from 1 to 5 mM, at room temperature [10]. Briefly, 10 mL of *T. cordifolia* leaf extract was added to 100 mL of an aqueous solution of 1 to 5 mM AgNO_3_ for the reduction of Ag into Ag+ ions. The aqueous leaf extract acted as a reducing agent as well as a stabilizing agent for the AgNO_3_. After a minimum of 30 min, the colorless solution changed to a transparent yellow color, and finally to a dark brown, indicating the formation of TcAgNPs. The reduction of the silver ions was monitored over time by UV–visible spectroscopy. Upon the formation of a dark brown color, the contents were centrifuged at 12,000 rpm for 20 min at 10 °C. The resultant pellet was gently washed with double-distilled water. Then, the extract was used for additional characterization.

### 2.3. Characterization of Green-Synthesized TcAgNPs

The TcAgNPs were characterized using the XRD Rigaku Miniflex with Cu Kα radians (D-max IIA, Rigaku, Japan). The UV-visible spectrophotometer (Shimadzu UV-1800, Kyoto, Japan) was used to investigate the optical properties of the TcAgNPs at 300 nm to 800 nm range wavelength [25]. Next using a Fourier transform infrared (FTIR) spectrophotometer (Perkin Elmer, Spectrum 2, Buckinghamshire, UK), the FTIR spectra were recorded using KBr discs with a wave number of 4000 cm^−1^ to 400 cm^−1^. The DLS was analyzed by the nanoparticle analyzer HORIBA SZ-100 (HORIBA, Ltd., Kyoto, Japan). The TcAgNPs (100 µg/mL) were suspended in DMSO (5% *v*/*v*), sonicated for 2 min, and the sample was processed in a nanoparticle analyzer. The analysis was repeated many times to obtain an accurate dynamic size of the nanoparticles. Zeta potential was measured using the same nanoparticle analyzer. Zeta potential depicts the nanoparticles’ stability and colloidal behavior, which can be measured in +/− mV [25]. The assay was performed in a solution containing 10 mM NaCl at pH 7.4, which closely mimics the biological conditions. The experiment was conducted three times, and each time repeated in triplicate, and mean electrophoretic mobility was −0.000167 cm^2^/Vs. Smoluchowski model was used to calculate the zeta potential in water-based solutions.

Next, the particle size, morphology and microstructure of TcAgNPs were examined by a high-resolution scanning electron microscopy (SEM) (JEOL, Ltd. E, Tokyo, Japan) [26]. The size, distribution, shape and morphology of TcAgNPs were evaluated with high resolution transmission electron microscopy (TEM) in a Jeol JEM 1400 HR (Joel Ltd., New Delhi, India) operated at an accelerating voltage of 120 kV at various magnifications. Briefly, 100 µg/mL of TcAgNPs (5% *w*/*v* DMSO) was subjected to ultrasonication for 5 min, and the sample was diluted 1:1 with distilled water. 20 µL of sample solution was added to the carbon coated copper grid and incubated for 10 min. The sample was deposited on a carbon coated copper grid for SEM-EDX analysis. Images were taken using an Emsis camera (EMSIS ASIA, Ltd., Burdwan, India) and were obtained using radius software. The size distribution of nanoparticle from TEM micrographs was analyzed using ImageJ software (Version 1.54p), and constructed the size distribution histogram.

Thermogravimetry analysis (TGA) was used to analyze the chemical composition of the leaf extract through volatiles analysis under sample heating. The assays were performed in a Thermo Scientific-iCE 3000 (Thermo Fisher Scientific, Waltham, MA, USA). About 2 mg of sample was heated up to 600 °C, under nitrogen flow of 50 mL min^−1^ and heating rate of 5 °C min^−1^. The equipment generates a TG curve of the sample. Analysis of the conversion of silver nitrate into AgNPs at different reaction times was examined with the atomic absorption spectrometer (AAS6300 Shimadzu, Kyoto, Japan).

### 2.4. Evaluation of Antioxidant Activity of TcAgNPs

#### 2.4.1. DPPH Radical Scavenging Activity

The DPPH (1,1-diphenyl-2-picrylhydrazyl) is a stable free radical commonly employed to assess the radical scavenging efficiency of antioxidants. DPPH free radical antioxidant assay method, which relies on electron transfer, yields a violet solution in methanol or ethanol. This free radical, stable at ambient temperature, is reduced by an antioxidant molecule, resulting in a colorless-ethanol solution. The radical scavenging or antioxidant activity of TcAgNPs was conducted by the DPPH assay using the Brand–William’s method [27]. The oxidized form of DPPH is a stable free radical with a purple-color activity that is inhibited by the electron donated by the anti-oxidant molecule. As a result, a change in absorbance at 517 nm can be observed using a UV-visible spectrophotometer, thereby assessing DPPH free radical scavenging activity.

First, 0.004% (*w*/*v*) of DPPH in methanol was prepared and stored in dark conditions. The DPPH solution was subjected to sonication using a probe homogenizer sonicator for 1 min at 5 s intervals. This sonication enables the stabilization of absorbance of the DPPH solution. In 1.5 mL micro centrifuge amber tubes, 600 µL of DPPH solution was taken. Stock solution of TcAgNPs was prepared as 1 mg/mL (5% DMSO *w*/*v*). The test sample with increasing concentrations of 25, 50, 75, and 100 µL of TcAgNPs were added to the DPPH solution into the microcentrifuge tubes. The reaction mixture was incubated in dark conditions for 40 min (room temperature), and the absorbance of the reaction mixture was read using UV-visible spectrophotometer at 517 nm wavelength. The radical scavenging activity (percentage) of TcAgNPs was calculated using the following formula:

Percent of radical scavenging activity = [(Ab_C_ − Ab_S_)/(Ab_C_)] × 100

Ab_C_—the absorbance of the DPPH solution, Abs—the absorbance of the TcAgNPs sample.

#### 2.4.2. ABTS Radical (2,2′-azino-bis (3-ethylbenzothiazoline-6-sulfonic Acid)) Scavenging Activity

ABTS°^+^ is a free radical assay based on the capacity of an antioxidant molecule to scavenge ABTS°^+^ radical cation, which is generated by the oxidation of ABTS with a potent oxidizing agent, such as potassium persulfate or potassium permanganate [28]. ABTS°^+^ radical cation is generated by mixing ABTS and potassium permanganate in water at a 1:0.5 ratio and allowing the reaction mixture to incubate in the dark for 16 to 24 h [29]. The ABTS+ solution is then diluted with water to obtain an absorbance reading of 0.734 ± 0.2 at 734 nm using a spectrophotometer.

First, 7 mM of ABTS was dissolved in 5 mL water. 2.45 mM of potassium permanganate was prepared for 5 mL. The ABTS°^+^ radical was generated by mixing ABTS solution with high oxidizing potential KMnO_4_ (2.45 mM) solution at a 1:0.5 ratio, i.e., for 5 mL of ABTS solution 2.5 mL of potassium permanganate solution was added. This reaction mixture was kept for 12 to16 h in dark conditions at room temperature. Before the assay, the reaction mixture was diluted with water till the absorbance was adjusted to 0.7 at 0.734 nm in the spectrophotometer. The stock solution of TcAgNPs was prepared at 1 mg/mL concentration. Then, 1 mL suspension of ABTS solution of 25, 50, 75, and 100 µL with concentrations and TcAgNPs of 25, 50, 75, and 100 µg/mL, respectively. The absorbance of the reaction mixtures incubated at room temperature for 20 min was read at 734 nm wavelength on a spectrophotometer [28]. The percentage inhibition was measured using the below formulae:

Percent inhibition = [(Ab_C_ − Ab_S_)/(Ab_C_)] × 100

Abc = absorbance of ABTS solution, Abs = absorbance of ABTS + TcAgNPs mixture.

### 2.5. Evaluation of Anti-Inflammatory Activity of TcAgNPs by Membrane Lysis Assay

Membrane lysis assay: Erythrocyte suspension was initially prepared as described by Shinde et al., [30] with minor modifications [31]. A fresh human blood specimen was obtained in a heparinized tube to inhibit coagulation. The blood sample was centrifuged at 10,000 rpm for 5 min. The resulted supernatant was discarded, and the pellet containing blood cells was gently washed thrice with 0.9% NaCl solution. Finally, the volume of the sample was reconstituted as 10% (*v*/*v*) in a phosphate buffered saline (1X PBS) and stored at 4 °C [32,33].

Heat-induced hemolysis: This assay was performed in accordance with the method of Gunathilake et al. [31]. For this assay, 50 µL of cell suspension and different concentrations of TcAgNPs 25, 50, 75, and 100 µL were added with 1 mL of 0.1 M sodium phosphate buffer (19% NaH_2_PO_4_ and 81% Na_2_HPO_4_). The contents were incubated for 30 min at 54 °C in a water bath. Following incubation, the contents were centrifuged at 10,000 rpm for 3–5 min, and discarded the supernatant. Optical density of the sample was read at 540 nm using a UV-visible spectrometer against a positive control (sodium phosphate buffer solution) for the experiment [27].

The percentage of protection was calculated using the following equation.

% protection = [(Ab_C_ − Ab_S_)/(Ab_C_)] × 100

Abc is the absorbance of the control containing only the reaction mixture and blood, and Abs is the absorbance/reading of the reaction mixture + TcAgNPs mixture.

### 2.6. Determination of Antibacterial Activity of TcAgNPs

In this study, we determined the antibacterial activity of TcAgNPs against multidrug resistance (MDR) bacteria by the disc diffusion method (DDM), the minimum inhibitory concentration (MIC), and the minimum bactericidal concentration (MBC) assays. The bacteria were revived from our previous work on MDR bacterial strains [10]. Bacterial strains were sub-cultured several times, and the pure culture was streaked onto a new plate and stored at 4 °C.

#### 2.6.1. Disc Diffusion Method

The TcAgNPs were tested for their antibacterial activity using the Kirby–Bauer DDM [34]. This assay was performed against the MDR bacterial strains, including *Pseudomonas aeruginosa*, *Klebsiella pneumonia*, *Escherichia coli*, and *Staphylococcus aureus* isolated from human wounds [35]. These bacterial strains were inoculated onto Mueller-Hinton Agar using a sterile L-rod. Antimicrobial susceptibility disc (HiMedia, Thane, India) was used as sterile blank. Ciprofloxacin (5 µg) was used as a positive control. The discs were infused with different concentrations of TcAgNPs. We used 5, 10, 15 and 20 μL volumes to load the discs with the corresponding concentrations of 25, 50, 75, and 100 µg/mL, respectively. The disc diameter is 10 mm, and the discs were placed on an agar plate, and incubated for 24 h at 37 °C. The clear zone of inhibition was observed following 24 h incubation.

#### 2.6.2. MIC and MBC Assays

The MIC as well as MBC were determined by the agar dilution method using INT (2-(4-Iodophenyl)-3-(4-nitro phenyl)-5-phenyl-2H-tetrazolium chloride) (HiMedia, India) as an indicator of growth. Inoculums of MDR bacterial strains were prepared according to the McFarlan standard, and then incubated for 24 h at 37° C. The bacterial broth was standardized to McFarlan standard 1.0, which is equivalent to 3.7 × 10^8^ cfu/mL for *E. coli*, 3.0 × 10^8^ cfu/mL for *K. pneumonia*, 3.2 × 10^8^ cfu/mL for *P. aeruginosa*; 3.0 × 10^8^ cfu/mL for *S. aureus* at 600 nm with 1.0 optical density value by using a spectrophotometer. The fresh broth was aliquoted in test tubes with 2 mL in volume. Then, 20 µL of each standardized bacterial strain was inoculated into the broth. The stock solution of TcAgNPs (5% DMSO *v*/*v*) at 1 mg/mL concentration with concentrations of 25, 50, 75, and 100 µg/mL, respectively, were added, then incubated for 18 h at 37 °C. Combination of Penicillin and Streptomycin used as a positive control with the same concentration as of TcAgNPs. INT 1 mg/mL dissolved in water, added to test tubes and incubated for 30 min at room temperature. Test tubes with bacterial growth turning from colorless to purple indicate no bacterial growth due to TcAgNPs [36]. The value of MIC was taken at the lowest concentration of the antibacterial agent that inhibit the bacterial growth.

MBC is interpreted as the test compound’s lowest concentration to kill bacteria completely. The MBC assay was carried out by plating the 20 µL suspension from each tube seeded on Nutrient agar plates (NAM). The agar plates were then incubated for 24 h at 37 °C. The minimal concentration with no visible/noticeable growth on the nutrient-agar plate was determined as MBC value [37].

### 2.7. Evaluation of Antibiofilm Activity of TcAgNPs

Antibiofilm assay tests the efficacy of potential antimicrobial agents against bacterial biofilms. In this assay, we used microtiter plate (96-well, flat bottom, polystyrene) and determined the antibiofilm efficacy of the TcAgNPs as explained by Gurunathan and colleagues [38] with minor modifications [39].

Each well of the plate was filled with 180 μL of Muller Hinton Broth. Then 10 μL of the test pathogen (*P. aeruginosa*, *K. pneumonia*, *E. coli*, and *S. aureus*, optical density = 1.0, 600 nm) was added, and incubated for a period of 16 h at 37 °C. TcAgNPs suspension (1 mg/mL in 5% DMSO) was loaded to each well at concentrations of 25, 50, 75, and 100 µg/mL, respectively, and again incubated for another 4–5 h at 37 °C. Penicillin and Streptomycin was used as a positive control with the same concentration as TcAgNPs. Broth without bacterial inoculum was used as a control. Following incubation, contents in the wells were discarded, and carefully washed with PBS (1X) to remove the non-adherent, free-floating bacterial cells from the bottom of each well. The microtiter plates were air-dried for 30 min. Following drying adherent “sessile” bacteria, 200 µL of crystal violet (0.1% *w*/*v*) was added, and incubated for 30 min in the dark. After 30 min, the wells were carefully washed with sterile distilled water to remove all excess dye, and air-dried again for 1 h. Upon drying, ethanol (200 µL) was loaded to each well, and absorbance was measured at 590 nm in a microplate reader [36]. The following equation was used to calculate the percentage of inhibition of biofilm formation [39].

Percent inhibition = [(Ab_C_ − Ab_S_)/(Ab_C_)] × 100

Ab_C_ is the absorbance of the control (only broth & bacteria), and Abs is the absorbance of bacterial broth with TcAgNPs.

### 2.8. Statistical Analyses

The obtained results were analyzed using the Graph pad prism, and origin 2018. The MIC and MBC were calculated by analysis of variance (ANOVA) employing descriptive statistics, such as means and standard deviations (SDs). Tukey’s post-hoc test was performed to analyze the MIC and MBC of AgNPs against MDR bacteria. Data were expressed as a mean ± SD for three replicates. Data considered statistically significance when *p* value is less than 0.05 (*p* < 0.05).

## 3. Results

### 3.1. Biosynthesis of TcAgNPs from T. cordifolia

**Figure 1:**
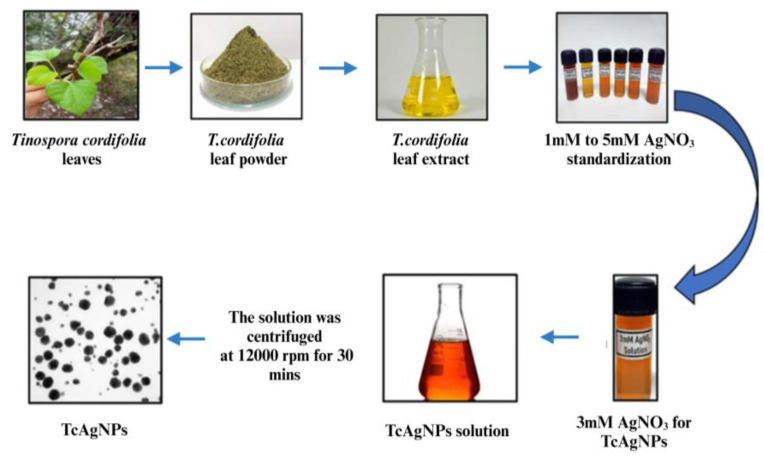
Pictorial illustration of green-synthesis of AgNPs from *Tinospora cordifolia* leaf extracts.

AgNPs were synthesized from *T. cordifolia* leaf extracts. We observed the gradual development of a brownish color after adding the leaf extracts to the 3 mM AgNO_3_ due to the bio-reduction of silver (Ag^+^) ions into silver NPs (AgNPs). Various concentrations of AgNO_3_ ranging from 1 to 5 mM combined with leaf extracts results the appearance of a darkish brown color, as illustrated in Figure 1, confirming the formation of TcAgNPs in all tested concentrations. The color change was observed with time interval 1 to 24 h. During the synthesis of TcAgNPs, the change of color from yellow-brown to darkish-brown due to the formation of AgNPs in the presence of biomass. Here, phytochemicals in leaf extract play an essential role in the biosynthesis of AgNPs. The color change in the suspensions is due to the TcAgNPs excitation with surface plasmon-resonance vibrations [40].

### 3.2. UV-Visible Spectra Analysis of TcAgNPs

**Figure 2:**
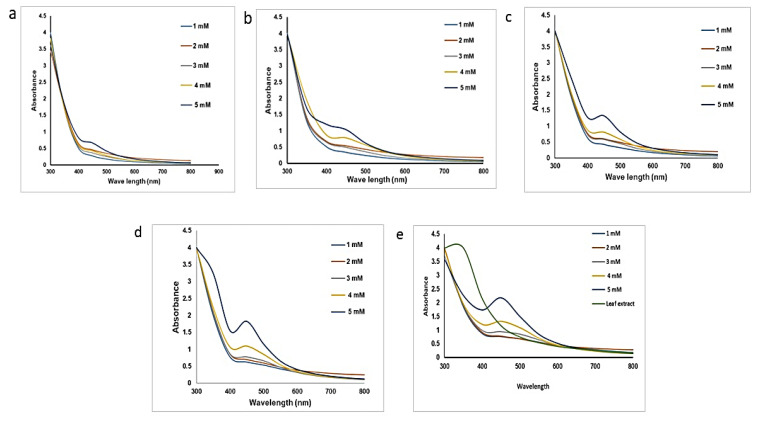
UV-visible spectroscopy of green-synthesized TcAgNPs at different time intervals represented as (**a**–**e**), at 2, 4, 6, 12, and 24 h respectively. (**e**) included with leaf extracts.

UV-visible spectral analysis determines the optical properties of the synthesized AgNPs as well as *Tc* leaf extracts. Several studies revealed that AgNPs exhibit an absorption peak around 412–470 nm [41,42]. The color change rate from yellow to darkish-brown was diverse in our studies, beginning within one hour to 24 h (Figure 2a–e). The samples were periodically observed in the UV-visible spectrophotometer at different concentrations of AgNO_3_ (1 to 5 mM) along with plant extract without AgNO_3_ during different time intervals at 2, 4, 6, 12, and 24 h. (Figure 2a–e). A sharp absorption band gradually increased from 1 to 24 h, while a 5 mM mixture showed maximum absorption peak after 24 h of incubation. The shift toward up reveals that the size of particle increased as the concentration increased. The curve sharpness was seen as the concentration increased due to the formation of the spherical to the cuboidal shape of the nanoparticles, as depicted in the UV-visible spectra graphs in Figure 2 [43]. Plant extracts exhibited a peak at 300, nm but no peak was seen between 400 nm and 500 nm (Figure 2e). The reduction of the Ag^+^ ions into AgNPs in the presence of leaf extracts can be noticed through the change in color. As the reaction progressed, the reduction occurred when the Ag^+^ ions captured an electron to become metallic silver Ag^0^. Data from the UV-visible spectroscopic results confirmed the formation of AgNPs from *Tc* leaf extracts.

### 3.3. X-Ray Diffraction Studies of TcAgNPs

**Figure 3:**
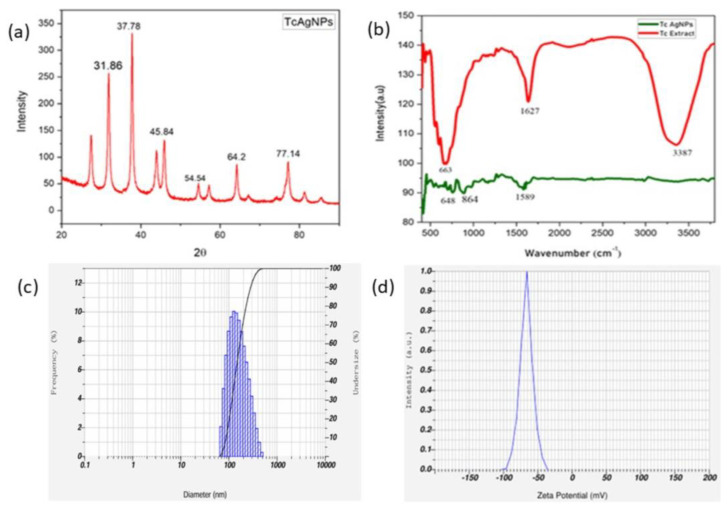
Structural characteristics of TcAgNPs using (**a**) XRD, (**b**) FTIR, (**c**) DLS (hydrodynamic size), and (**d**) zeta potential analyses.

TcAgNPs were characterized using XRD with the Rigaku Miniflex. The XRD analysis validated the crystalline characteristics of the green-synthesized TcAgNPs (Figure 3a). The graph reflects the patterns of the face-centered cubic (fcc) and crystalline structure of the biosynthesized AgNPs. The peaks of the (200) and (311) planes were noticed in the AgNPs biosynthesized by the *Convolvulus arvensis* extracts [44]. The size of the nanoparticles significantly influences the XRD peak patterns [45].

### 3.4. FTIR Analysis of TcAgNPs

The FTIR technique was used to measure the secondary metabolites involved in the synthesis of the AgNPs. The FTIR spectra of multiple peaks of *T. cordifolia* leaf extract showed significant and minor peaks, acting as reducing and capping agents, and stabilizing the nanoparticles during the synthesis of TcAgNPs. The characteristic FTIR spectrum, as depicted in Figure 3b, showed peaks at 3387, 1627, and 663 cm^−1^ in leaf extracts, and 1589, 864, and 648 cm^−1^ in the AgNPs. In the spectrum, a sharp absorption peak at 3387 cm^−1^ corresponds to the N-H bonds of amino groups of alcoholic and phenolic compounds of *T. cordifolia*, stabilizing the TcAgNPs [43]. The presence of N–H group-specific proteins and enzymes corresponds to the reduction of AgNO_3_ to Ag+. The visible peak at 1627 cm^−1^ in the TcAgNPs depicts the existence of C=C functional groups of proteins and metabolites in the plant extract [46]. The peaks between 663 cm^−1^ and 648 cm^−1^ are due to C-Cl alkyl halide stretching [47]. Most of the peaks resemble the phenolic group of triterpenoids, alkaloids, steroids, and polyphenols. The existence of these compounds in the leaf extracts helps to form TcAgNPs.

### 3.5. DLS and Zeta Potential of TcAgNPs

The silver solution was diluted to reduce scattering. The hydrodynamic diameter, particle size, and polydispersity index (PDI) of the samples were measured using DLS. The size and distribution graph reveals the average size of the TcAgNPs, approximately 168 nm (Figure 3c). The PDI is 0.26, indicating that the silver solution was profusely dispersed in a liquid medium. Zeta potential can be used to study the electric potential at the shear plane surrounding the nanoparticles in a dispersion medium. Zeta potential greater than +30 mV or less than −30 mV shows higher stability of the nanoparticles [48]. In our study, the zeta potential of the TcAgNPs was −32.3 mV. This negative zeta potential illustrates stronger electrostatic repulsion among the nanoparticles and greater stability [25,49]. The green-synthesized nanoparticles exhibited greater stability in suspension, as depicted in Figure 3d. Hence, electrostatic repulsion of the green-synthesized AgNPs was sustained with great stability in water. The results of the zeta potential analysis suggest that green-synthesized TcAgNPs exhibit greater stability and resist agglomeration.

### 3.6. SEM Assessments of TcAgNPs

**Figure 4:**
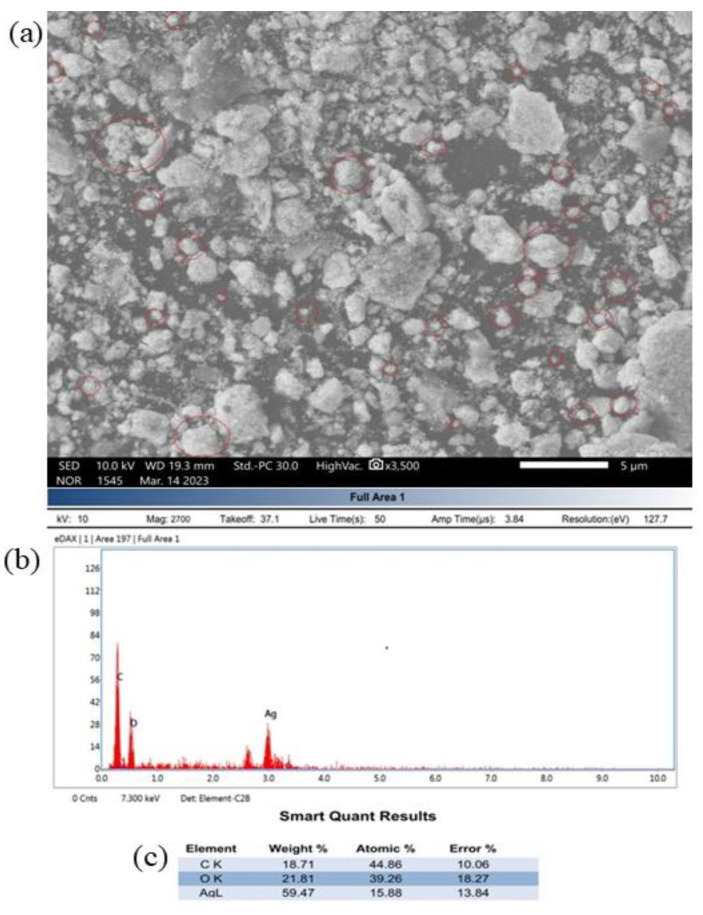
Structural characteristics of TcAgNPs: (**a**) scanning electron microscope image: spherical structures of TcAgNPs are labeled with a red outline; (**b**) energy dispersive X-ray spectrum showing the presence of silver; (**c**) elemental composition of TcAgNPs from EDX analysis.

SEM was used to determine the morphology of the synthesized AgNPs [50]. The images reveal the near-spherical structure and shape of the TcAgNPs (Figure 4a upper panel). The nanoparticles in the images are outlined in red to indicate their shape. The EDX spectrum (Figure 4b, lower panel) reveals the occurrence of silver elemental signals, confirming that the particles are TcAgNPs produced through biosynthesis from *T. cordifolia* with AgNO_3_. The x- and y-axes represent the number of X-ray counts and the energy in keV, respectively. The sharp elemental signal peaks indicate the significant emission energies for silver, and the lines in the EDX spectrum demonstrate that silver was identified. The silver peak was derived from the TcAgNPs, and its atomic percentage in the TcAgNPs was 15.88%, while the atomic percentages of carbon (C) and oxygen (O) were 44.86% and 39.26%, respectively (Figure 4c). Carbon is a fundamental element in the phytochemistry of *T. cordifolia*. The SEM-EDX analysis was carried out with dry-state powdered TcAgNPs, and there was agglomeration, reflecting its higher percentage in the EDX results. Other than carbon and oxygen, the remaining elements showed very low atomic percentages in the EDX spectrum. The SEM analysis coupled with the EDX spectrum revealed the synthesis of nanoparticles from leaf extracts of *T. cordifolia* using AgNO_3_ as a metal precursor to produce TcAgNPs.

### 3.7. TEM Assessments of TcAgNPs

**Figure 5:**
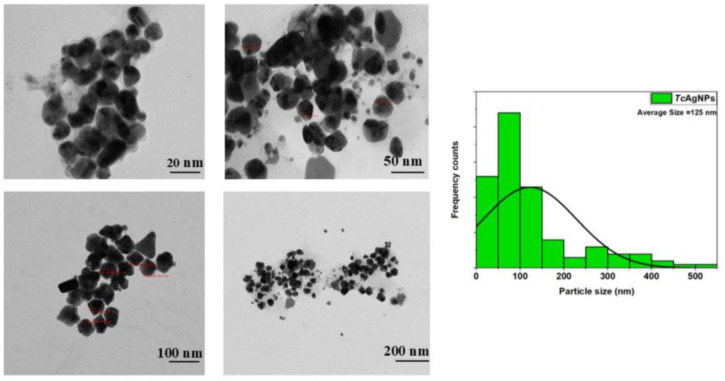
TEM images and particle size distribution histogram of TcAgNPs at different magnifications.

Our findings demonstrated that the green-synthesized silver nanoparticles were nearly spherical. The TEM micrographs revealed variations in particle size, and the average particle size was 125 nm, as depicted in Figure 5. Furthermore, the average particle size distribution was analyzed using ImageJ software, and the histogram showed the size distribution range of the TcAgNPs at different magnifications (Figure 5). Most of the particles had the same spherical shape, showing that the TcAgNPs were monodisperse, with greater stability, in agreement with their zeta potential [40].

### 3.8. Thermogravimetry of TcAgNPs

**Figure 6:**
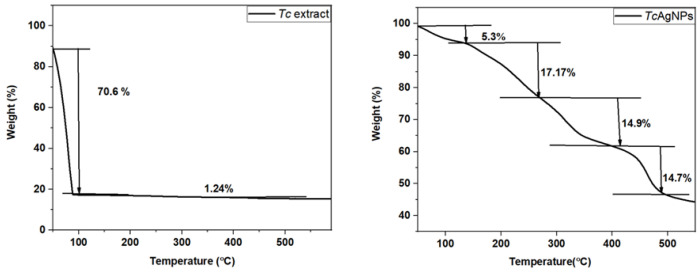
TGA thermograph of *T. cordifolia* leaf extracts and TcAgNPs.

Thermogravimetric analysis of *T. cordifolia* leaf extracts and green-synthesized TcAgNPs was performed by altering the temperature from 0 to 600 °C, as indicated in Figure 6. In the first stage, the TGA curve of the plant extract illustrated weight loss with an increase in temperature to 100 °C, due to the evaporation of the water content present in the *T. cordifolia* extracts [51]. In the second stage, with an increase in the temperature to above 500 °C, almost no alteration in weight loss was noticed. From the TGA curve, notable weight loss of AgNPs can be seen at temperatures between 250 °C to 300 °C. A small weight loss occurred below 350 °C and above 400 °C [52]. The initial decomposition occurred from room temperature to 160 °C, with 5.3% weight loss, which indicates the vanishing of water-containing impurities from the TcAgNPs. In the second stage, with the temperatures ranging from 200 to 300 °C, about 17.17% weight reduction was observed, representing the decomposition of capping organic compounds (including carbonyl and reducing sugar) acting as the core shell surface of nanoparticles. Next, the third and final step led to the decomposition of TcAgNPs at higher temperatures between 400 °C and 600 °C, where the weight loss was 14.9 and 14.7%, respectively. This particular weight loss of AgNPs may be related to their assembly, which enhances the decomposition of the leaf extract contents. Nevertheless, the weight loss did not approach zero, due to the presence of AgNPs in TcAgNPs.

### 3.9. Atomic Absorption Spectroscopy of TcAgNPs

**Figure 7:**
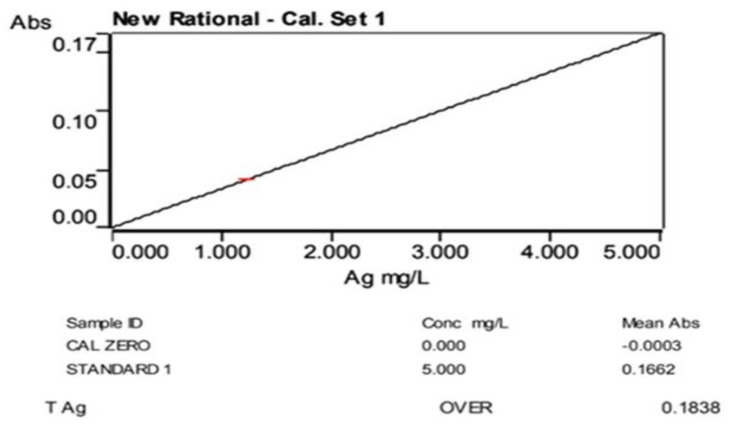
Silver concentration (ppm) in green-synthesized silver nanoparticles.

The concentrations of silver ions in the green-synthesized silver nanoparticles were determined using AAS. A standard solution of 1 to 5 ppm of AgNO_3_ was initially prepared and analyzed by AAS at 0 min. The silver ion concentration in the reaction solution was determined after the addition of TcAgNPs. The findings indicated an increase in Ag ion concentration from 1 to 5 ppm at an absorbance of 0.05 to 0.17, respectively. These results imply the conversion of Ag ions into AgNPs. We constructed a calibration curve, as shown in Figure 7. The curve has good linearity in a concentration range of about 5.529 ppm at 5 mg of TcAgNPs. The data revealed that 90% of silver nitrate was converted into silver nanoparticles.

### 3.10. TcAgNPs Exhibit Free Radical Scavenging Activity

**Figure 8:**
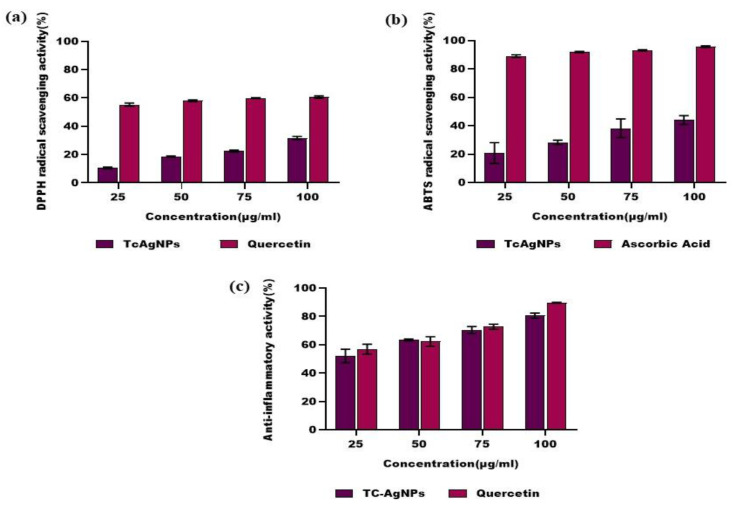
Antioxidant properties of different concentrations of TcAgNPs: (**a**) DPPH radical scavenging activity and (**b**) ABTS radical scavenging activity. (**c**) Anti-inflammatory activity of different concentrations of TcAgNPs. Values expressed as mean ± standard deviation.

The free radical scavenging or antioxidant activity of the TcAgNPs was evaluated by the DPPH radical scavenging assay. The TcAgNPs exhibited DPPH radical scavenging abilities in a concentration-dependent manner, as represented in Figure 8a. The DPPH radical scavenging abilities of the TcAgNPs were increased with increased concentrations of TcAgNPs. A well-known antioxidant (quercetin) was used as a control [53]. The free radical scavenging activity of the green-synthesized TcAgNPs might be associated with the presence of bioactive compounds on the surface of the NPs, as well as their tendency to donate hydrogen atoms to free radicals. The DPPH radical scavenging abilities of the TcAgNPs were 10, 18, 22, and 31% at concentrations of 25, 50, 75, and 100 µg/mL, respectively.

Next, we determined the antioxidant activity of the TcAgNPs by the ABTS radical scavenging assay, using ascorbic acid as a control. The ABTS radical scavenging abilities of the TcAgNPs are represented in Figure 8b. The ABTS radical scavenging abilities of the TcAgNPs were 20, 28, 38, and 44% at concentrations of 25, 50, 75, and 100 µg/mL, respectively.

### 3.11. Anti-Inflammatory Efficacy of TcAgNPs

The anti-inflammatory properties of the TcAgNPs were evaluated by membrane lysis assays, and the results are presented in Figure 8c. The TcAgNPs exhibited a concentration-dependent anti-inflammatory ability, and the percentage protection was increased with increasing concentrations of TcAgNPs [32]. Quercetin was used as a control, and the anti-inflammatory efficiency of the TcAgNPs was similar to that of quercetin. The protection of the TcAgNPs was 52, 63, 70, and 80% at concentrations of 25, 50, 75, and 100 µg/mL, respectively.

### 3.12. TcAgNPs Shows Potent Antibacterial Activity Against MDR Bacteria

The antibacterial activity of the TcAgNPs against various strains of MDR bacteria was evaluated by the DDM, MIC, and MBC assessments.

#### 3.12.1. Disc Diffusion Method

**Figure 9:**
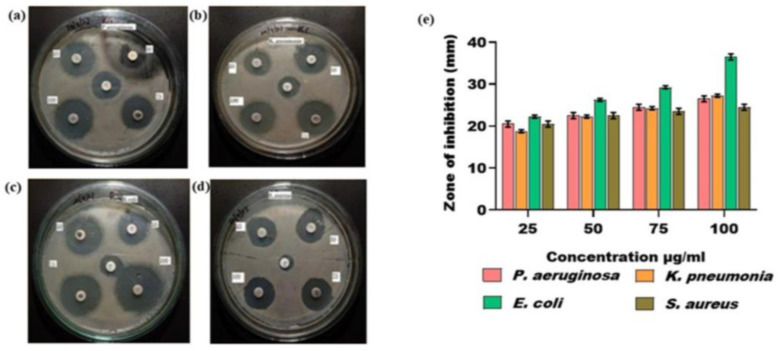
Zones of inhibition with different concentrations of TcAgNPs against multidrug-resistant (MDR) bacteria: (**a**) *Pseudomonas aeruginosa*, (**b**) *Klebsiella pneumonia*, (**c**) *Escherichia coli*, and (**d**) *Staphylococcus aureus*. (**e**) Graphical presentation of zones of inhibition of TcAgNPs against MDR bacteria. Values are expressed as the mean ± standard deviation.

The findings from the DDM showed effective antibacterial activity of the TcAgNPs against MDR bacteria (*P. aeruginosa*, *K. pneumonia*, *E. coli,* and *S. aureus*) that were isolated from wound infections. Ciprofloxacin (5 µg) antibiotic discs were used as a standard. As shown in Figure 9a,b (Table S1), the TcAgNPs showed potent antibacterial properties against the Gram-negative (*P. aeruginosa*, *K. pneumonia*, and *E. coli*) and Gram-positive bacteria (*S. aureus*), as evidenced by a broadening inhibitory zone with increasing concentrations (25, 50, 75, and 100 µg/µL). The maximum antibacterial activity of the TcAgNPs was shown as a 37 mm zone of inhibition at the highest concentration against *E. coli*. Other studies have also reported similar results with green-synthesized silver nanoparticles [12,23].

#### 3.12.2. MIC and MBC of TcAgNPs Against MDR Bacteria

**Figure 10:**
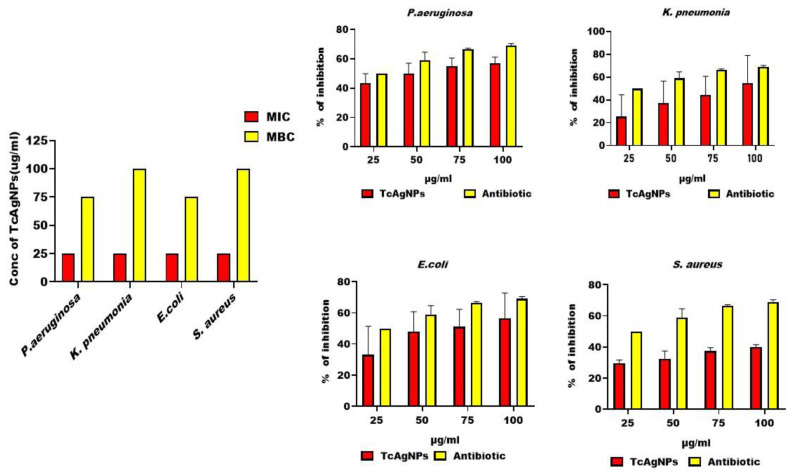
Antibiofilm activity with different concentrations of TcAgNPs: (**a**) MIC and MBC efficacy of TcAgNPs. (**b**) Percentage inhibition ability of TcAgNPs and antibiotic against biofilm formation of *P. aeruginosa*, *K. pneumonia*, *E. coli*, and *S. aureus.* Values are expressed as the mean ± standard deviation.

The MIC of the TcAgNPs against MDR bacteria was evaluated using the dilution method. A penicillin and streptomycin combination antibiotic was used as a positive control. After a 12 h incubation in aerobic conditions, no turbidity was noticed in the test tubes at any concentration of TcAgNPs (25, 50, 75, and 100 µg/m). In addition, no visible color change was noticed in the bacterial broth after adding INT (2-(4-Iodophenyl)-3-(4-nitro phenyl)-5-phenyl-2H-tetrazolium chloride) as a growth indicator. There was a visible color change in the antibiotic (penicillin plus streptomycin)-treated samples, showing their multidrug resistance, as shown in Table S1. The MIC of the TcAgNPs against all MDR bacteria (*P. aeruginosa*, *K. pneumonia*, *E. coli*, and *S. aureus*) was 25 µg/mL (Figure 10a).

To determine the MBC, the assay was followed by taking samples ranging from no visible color change to color change after adding INT. After observing no color change in the bacterial broth at concentrations of 25, 50, 75, or 100 µg/mL TcAgNPs, treated samples were streaked onto agar plates to determine the ability of TcAgNPs to kill the bacteria. Bacterial growth was observed in all plates with all concentrations of TcAgNPs (25, 50, 75, and 100 µg/mL) (Figure 10a). In plates containing *E. coli* and *K. pneumonia*, no bacterial growth was observed only at the concentration of 100 µg/mL, which depicts the minimum concentration needed for the bactericidal properties of TcAgNPs. In contrast, at a 100 µg/mL antibiotic concentration, the MDR *E. coli* did not show growth, in agreement with the MDR nature of the bacteria, as depicted in Tables S2–S4.

### 3.13. Antibiofilm Activity of TcAgNPs

The antibiofilm activity of the TcAgNPs was evaluated against MDR bacteria by the microplate method. Each bacterial species was individually grown in a 96-well microtiter plate for 16 h and treated with various concentrations of TcAgNPs (25, 50, 75, and 100 µg/mL) in each well. The TcAgNPs treatment showed a concentration-dependent antibiofilm activity against MDR bacteria, including *P. aeruginosa*, *K. pneumonia*, *E. coli*, and *S. aureus*. The biofilm inhibition abilities of the tested concentrations of TcAgNPs against *P. aeruginosa* were 41, 50, 57, and 58%, respectively. Similarly, the biofilm inhibition percentages with these TcAgNPs concentrations (25, 50, 75, and 100 µg/mL) against other MDR strains were as follows: *K. pneumonia*, 22, 36, 42, and 55%, respectively; E. coli, 32, 44, 52, and 57%, respectively; *S. aureus*, 30, 33, 38 and 39%, respectively (Figure 10b).

## 4. Discussion

*T. cordifolia* is renowned for its medicinal uses. However, only a few studies have emphasized the pharmacological efficiency of metal nanoparticles synthesized from *T. cordifolia* leaf extracts. Our study demonstrated successful synthesis and structural characterization of AgNPs from *T. cordifolia* leaf extracts. Using advanced techniques, we determined the shape, size, size distribution, morphology, stability, and structural properties of the TcAgNPs. Furthermore, these biosynthesized *T. cordifolia* AgNPs exhibited free radical scavenging, anti-inflammatory, antimicrobial, and antibiofilm activities. The technique of green synthesis is non-toxic, cost-effective, and more stable compared to other biological methods.

The bio-reduction of silver ions to silver NPs was affirmed by a gradual color change of the reaction mixture from yellow-brown to dark brown, as observed in the UV–visible spectroscopy absorbance from 412 to 470 nm. The XRD results indicated the crystalline nature of the synthesized TcAgNPs, which is consistent with earlier findings [54]. The FTIR analysis showed the presence of various functional groups that act as reducing and capping agents. The distinct peaks from the FTIR plot reveal the existence of O-H and N-H bonds of amino groups of alcoholic and phenolic compounds; N–H group-specific proteins and enzymes; amide C=O, C=C, and CH3 aromatic groups; alkenes and alkynes; and groups of proteins and metabolites. Using DLS, the hydrodynamic diameter, typical particle size, and PDI of the TcAgNPs were measured, and the average particle size was 36.6 nm. The stability, dispersion, and surface charge of the TcAgNPs were evaluated by zeta potential, which was −66.7 mV, owing to the stable nature of the TcAgNPs. These findings are in agreement with previous reports [48,50]. The SEM-EDX revealed the presence of the elements silver, carbon, and oxygen. The TEM data showed that the average size of the nanoparticles was 125 nm. Both the SEM and TEM results are consistent with previous studies described by Rauwel and colleagues [55]. The TGA results suggest the complete decomposition of the TcAgNPs due to the desorption of bioactive organic compounds. The desorption process occurred as a result of the interaction between bioactive/volatile organic compounds in the leaf extract and the surface of the synthesized nanoparticles [56].

We then determined the antioxidant properties of the TcAgNPs at various concentrations. Our data indicated that the TcAgNPs exhibited promising antioxidant activity, in a concentration-dependent manner. DPPH (oxidized form) activity is inhibited by the electron donated by the antioxidant molecule. Here, the TcAgNPs showed a potent radical scavenging ability compared with the standard antioxidant quercetin. Kanagala et al. synthesized AgNPs from the ripe and unripe fruit extracts of *T. cordifolia*, and they reported greater free radical scavenging efficacy and antibacterial activity [52]. Another study synthesized selenium NPs from the stems of *T. cordifolia* and showed higher DPPH scavenging activity with higher concentrations of synthesized NPs [57]. ABTS°^+^ is a free radical that is scavenged by an antioxidant molecule by donating electrons, producing an ABTS radical [28]. A recent study reported that *T. cordifolia* leaf extracts could serve as a reducing agent owing to their high phenolic, flavonoid, alkaloid, and sterol contents [5]. In our study, TcAgNPs showed significant radical scavenging activity, comparable to that of ascorbic acid, a standard antioxidant. These findings emphasize the efficiency of TcAgNPs in trapping and scavenging the free radicals. We assume that the phytochemicals, phenolic compounds, aromatic groups, and metabolites in the leaf extracts might be responsible for the effective free radical scavenging ability of the TcAgNPs. However, it is inconclusive whether the free radical scavenging activity of the TcAgNPs was due to the presence of phytochemicals, silver in the nanoparticles, or the combination of phytochemicals and silver.

Another novel finding of our study is that various concentrations of TcAgNPs showed significant anti-inflammatory activity. We used the membrane lysis assay to determine the anti-inflammatory activity of the TcAgNPs, which acted as a protective agent against the external stress simulated on the human RBCs [31,32,33]. It has been stated that protecting cells from external stress demonstrates the anti-inflammatory properties of the compounds [5]. The reported anti-inflammatory properties in our study were comparable with those of the standard anti-inflammatory agent quercetin. Similar to our findings, Prakash and colleagues also reported antioxidant and anti-inflammatory properties of copper NPs from *T. cordifolia* leaves [58]. Evidence showed that the pre-treatment of cells with chloroform extracts of *T. cordifolia* significantly suppressed the release of pro-inflammatory cytokines against lipopolysaccharide (LPS) induction [59]. A recent study has shown a potent anti-inflammatory effect of green-synthesized TcAgNPs against LPS-induced testicular inflammation in golden hamsters [60]. Although we do not have molecular evidence or in vivo data to validate the anti-inflammatory properties of TcAgNPs, the phytochemicals present in the TcAgNPs and their free radical scavenging activity might have contributed to the anti-inflammatory effect.

Since MDR bacteria are deadly pathogens, the spread of these bacteria in society ultimately increases the risk of antibiotic use, morbidity, and mortality [24]. Therefore, studies on how to kill MDR bacteria or inhibit their growth, particularly with plant extracts and NP treatments, are crucial, as they offer potential strategies to treat MDR-associated diseases. To provide strong evidence and to emphasize the clinical significance of *T. cordifolia*, the antibacterial efficacy of the TcAgNPs against MDR bacteria was determined by DDM, MIC, MBC, and antibiofilm assays. The findings from the DDM showed notable antibacterial activity (zone of inhibition) with all concentrations of TcAgNPs (25, 50, 75, and 100 µg/µL) against all types of MDR bacteria (*P. aeruginosa*, *K. pneumonia*, *E. coli*, and *S. aureus*). Phanse et al. [23] examined the antibacterial efficacy of only one concentration of TcAgNPs against *S. aureus*, *P. aeruginosa*, *S. typhi*, and *E. aerogenes*, and the reported bacterial inhibition zones were not greater than those in our study. Another study reported that the maximum zone of inhibition with TcAgNPs (10 mg/mL) against *Staphylococcus* and *Klebsiella* species was 13 mm and 12.3 mm, respectively [12]. For the first time, we used various concentrations of TcAgNPs, and the zone of inhibition against all MDR bacteria (*P. aeruginosa*, *K. pneumonia*, *E. coli*, and *S. aureus*) was about 20 nm. Similarly, the results from the MIC and MBC assays also revealed promising antibacterial activity of the TcAgNPs, in a concentration-dependent manner. These findings explain the efficacy of TcAgNPs in the inhibition of bacterial growth, while promoting bacterial death at the tested concentrations.

Although previous studies have reported the antibacterial activity of *T. cordifolia* leaf extracts and their NPs [5,12,23], the reported antibacterial activity in these studies was not against MDR bacterial strains. Furthermore, biofilms are associated with many health problems, especially in the field of medicine. Biofilms bind to various medical devices, leading to persistent infections. Another novel finding of our study is the potent antibiofilm activity of TcAgNPs. Among the four bacterial strains tested here, *S. aureus* showed the lowest inhibition percentage with all concentrations of TcAgNPs. This might have been due to bacteria targeting the weak points over the colloidal dispersion of TcAgNPs. The aggregate stability was weak due to the environmental and physical conditions of the TcAgNPs, and repeated exposure of *S. aureus* to TcAgNPs also results in a lower inhibition rate [5,61]. The use of NPs could be a safe and non-toxic approach to eradicating biofilms [62]. The standard deviations of antibiofilm activity, particularly for *K. pneumonia* and *E. coli*, were slightly higher with AgNPs. Factors such as the sample size or the bacterial status in the sample may have contributed to higher SDs.

AgNPs have shown efficient antibacterial properties by penetrating the cell, where they bind to the sulfur or phosphorus of the protein or DNA, leading to destabilization of the bacterial cell irrespective of its drug-resistant nature [63]. It has been shown that green-synthesized NPs from plants extract exhibit significant antibacterial activity against MDR bacteria [64]. Das et al. showed that AgNPs possess potent antibiofilm activity against MDR bacteria [65]. Continuous release of silver ions from AgNPs in an aqueous microenvironment contributes to their antibacterial activity. Typically, NPs have a small volume and large surface area, helping them to penetrate the cell membrane, causing a disturbance in the membrane permeability of the cell, making it porous, and causing further leakage of the cell constituents [66,67,68], Another hypothesis suggests that Ag+ released from AgNPs through the oxidation process promotes the production of reactive oxygen species (ROS), which then oxidize the bacterial cell membrane and cause cell death [5,66]. Green-synthesized and cost-effective AgNPs from other plant extracts have also displayed potent antibacterial activity [69,70]. Taken together, the various concentrations of TcAgNPs in our study were found to have potent antibacterial activity, which could enable their use in the medical field upon further confirmatory studies.

### Limitations

Despite the well-established structural characteristics of TcAgNPs, determined with advanced techniques, there are few limitations in our study. The antibacterial, anti-inflammatory, antioxidant, and antibiofilm activities were reported with TcAgNPs, not with *T. cordifolia* leaf extract alone. In is inconclusive whether the antibacterial, antioxidant and anti-inflammatory effects of the AgNPs were due to the presence of silver, to the presence of the plant extract, or the combination (synergistic effect) of both. The reported pharmacological properties were supported by the in vitro experiments only; in vivo testing on animal models remains to be conducted.

## 5. Conclusions

Our findings concluded that the green-synthesized TcAgNPs possessed potent free radical (DPPH/ABTS) scavenging activity and anti-inflammatory activity, in a concentration-dependent manner (25–100 µg/mL). Similarly, the TcAgNPs showed significant antibacterial activity against multidrug-resistant bacteria (*P. aeruginosa*, *K. pneumonia*, *E. coli*, and *S. aureus*), which is a novel finding. The antibiofilm activity of the TcAgNPs against MDR bacterial stains further emphasizes the pharmacological value of TcAgNPs. These novel findings emphasize the importance of the studied concentrations of TcAgNPs (25–100 µg/mL), which could be used in the development of potential antioxidant, anti-inflammatory, and antibacterial substances.

## Data Availability

All of the original data of this study are available from the corresponding authors upon request.

## Supplementary Materials

The following supporting information can be downloaded at https://www.mdpi.com/article/10.3390/nano15050381/s1: Table S1: MIC of TcAgNPs against MDR bacteria; Table S2: MIC of antibiotics against MDR bacteria; Table S3: MBC of TcAgNPs against MDR bacteria; Table S4: MBC of antibiotics against MDR bacteria.
