# Supplementary material for: Green Synthesis and Characterization of Silver Nanoparticles from Tinospora cordifolia Leaf Extract: Evaluation of Their Antioxidant, Anti-Inflammatory, Antibacterial, and Antibiofilm Efficacies
Source: Nanomaterials (Basel). 2025 Mar 1;15(5):381. doi: 10.3390/nano15050381 (PMC11901609; doi:10.3390/nano15050381)
Supplement: Supplementary file 1 [file nanomaterials-15-00381-s001.zip › nanomaterials-3399959-supplementary.pdf]

Supplementary Tables S1 to S4

Table S1. Minimum inhibitory concentration (MIC) of TcAgNPs against (a) *P. aeruginosa* (b) *K. pneumonia,* (c) *E. coli*, and (d) *S. aureus.* Positive (+): turbidity, indicating growth. Negative (-): no turbidity, indicating absence of growth.

1. 2. Bacterial strains	3. Color change in culture broth after adding INT at various concentrations of TcAgNPs for evaluating MIC			
	4. 25 µg/mL	5. 50 µg/mL	6. 75 µg/mL	7. 100 µg/mL
8. *P. aeruginosa*	9. -	10. -	11. -	12. -
13. *K. pneumonia*	14. -	15. -	16. -	17. -
18. *E. coli*	19. -	20. -	21. -	22. -
23. *S. aureus*	24. -	25. -	26. -	27. -

Table S2. Minimum inhibitory concentration (MIC) of antibiotic against (a) *P. aeruginosa*, (b) *K. pneumonia*, (c) *E. coli*, and (d) *S. aureus*. Positive (+): turbidity, indicating growth. Negative (-): no turbidity, indicating absence of growth.

Bacterial strains	Color change in culture broth after adding INT at various concentrations of penicillin and streptomycin for evaluating MIC			
	25 µg/mL	50 µg/mL	75 µg/mL	100 µg/mL
P. aeruginosa	+	+	+	+
K. pneumonia	+	-	+	+
E. coli	-	+	+	+
S. aureus	+	+	+	-

Table S3. Minimum bactericidal concentration (MBC) of TcAgNPs against (a) *P. aeruginosa*, (b) *K. pneumonia*, (c) *E. coli*, and (d) *S. aureus*. Positive (+): turbidity, indicating growth. Negative (-): no turbidity, indicating absence of growth.

Bacterial strains	Bacterial growth on agar plate at various concentrations of TcAgNPs for evaluating MBC			
	25 μg/mL	50 μg/mL	75 μg/mL	100 μg/mL
P. aeruginosa	+	+	+	-
K. pneumonia	+	+	+	+
E. coli	+	+	+	-
S. aureus	+	+	+	+

Table S4. Minimum bactericidal concentration (MBC) of antibiotic against (a) *P. aeruginosa*, (b) *K. pneumonia*, (c) *E. coli*, and (d) *S. aureus*. Positive (+): turbidity, indicating growth. Negative (-): no turbidity, indicating absence of growth.

Bacterial strains	Bacterial growth on agar plate at various concentrations of penicillin and streptomycin for evaluating MBC			
	25 μg/mL	50 μg/mL	75 μg/mL	100 μg/mL
P. aeruginosa	+	+	+	+
K. pneumonia	+	+	+	+
E. coli	+	+	+	-
S. aureus	+	+	+	+
